# Socio-Cultural Determinants of Health-Seeking Behaviour on the Kenyan Coast: A Qualitative Study

**DOI:** 10.1371/journal.pone.0071998

**Published:** 2013-11-18

**Authors:** Amina Abubakar, Anneloes Van Baar, Ronald Fischer, Grace Bomu, Joseph K. Gona, Charles R. Newton

**Affiliations:** 1 Utrecht University, Utrecht, The Netherlands; 2 Centre for Geographic Medicine Research-Coast, KEMRI, Kilifi, Kenya; 3 Tilburg University, Tilburg, The Netherlands; 4 Victoria University of Wellington, Wellington, New Zealand; 5 University of Oxford, Oxford, United Kingdom; Edinburgh University, United Kingdom

## Abstract

**Background:**

Severe childhood illnesses present a major public health challenge for Africa, which is aggravated by a suboptimal response to the child's health problems with reference to the health-seeking behaviour of the parents or guardians. We examined the health-seeking behaviour of parents at the Kenyan coast because understanding impediments to optimal health-seeking behaviour could greatly contribute to reducing the impact of severe illness on children's growth and development.

**Methods and Results:**

Health-seeking behaviour, and the factors influencing this behaviour, were examined in two traditional communities. We held in-depth interviews with 53 mothers, fathers and caregivers from two rural clinics at the Kenyan Coast. Biomedical medicine (from health facilities and purchased over the counter) was found to be the most popular first point of treatment. However, traditional healing still plays a salient role in the health care within these two communities. Traditional healers were consulted for various reasons: a) attribution of causation of ill-health to supernatural sources, b) chronic illness (inability of modern medicine to cure the problem) and c) as prevention against possible ill-health. In developing an explanatory model of decision-making, we observed that this was a complex process involving consultation at various levels, with elders, but also between both parents, depending on the perceived nature and chronicity of the illness. However, it was reported that fathers were the ultimate decision makers in relation to decisions concerning where the child would be taken for treatment.

**Conclusions:**

Health systems need to see traditional healing as a complementary system in order to ensure adequate access to health care. Importantly, fathers also need to be addressed in intervention and education programs.

## Introduction

Each year, millions of children die of preventable and treatable conditions - largely in low- and middle-income countries - because they do not access biomedical treatment in a timely manner [1]. Among those who survive early severe ill-health, there is documented impairment in cognitive and social emotional functioning which can persist, causing a significant impact on the individual, the household and the community [2], [3]. The lack of timely and adequate medical care is a significant contributor to mortality and morbidity in these resource-poor settings. Inadequate financial resources and an under-resourced health care system contribute to delay in accessing medical facilities. These two cardinal factors only partially explain the observed trends in low uptake of optimal treatment patterns [4]. Earlier studies indicate that a range of other factors - such as the relatively low status of women [5], cultural beliefs and practices [6], and perception of the cause of the illness [7] - may contribute to this delay for parents to access medical care for their children. Observations indicate that if parents perceive a certain illness to be unrelated to biomedical causes, they are less willing to go for biomedical care, or at the very least, may delay the speed at which they take up biomedical care [4]. These observations point to the central role of socio-cultural factors in determining health-seeking behaviour.

Previous research in Africa has investigated parental perceptions of the aetiology of different diseases, and their health-seeking behaviour. The focus has largely been on describing socio-cultural determinants of health-seeking behaviour in the context of specific health conditions; but these studies have largely focussed on malaria [8], [9], malnutrition [5], [10] and epilepsy [11]–[13]. However, there is a need to take a non-disease-specific perspective, since in certain cases some health-related beliefs and practices may be pervasive and may influence health-seeking behaviour in different contexts. A good example of this is provided by the issue of *Abiku* among the Yoruba [7]. *Abiku* refers to a belief that some children are from the spirit world, and will die at will. Consequently, some mothers recommend that the care for these children would be different from that of normal children. With no specific set of syndromes , the diagnosis of *abiku* is largely based on the child's non-response to treatment (both biomedical and traditional) and upon confirmation by traditional healers [14]. This belief in a ‘spirit child’ may influence the treatment of children with different chronic illnesses. Consequently, investigation of health-seeking behaviour may require a non-disease- specific approach.

We focussed on the general conceptualisation of ill-health and management of disease through identification of common themes across different childhood diseases. The strength of this approach lies in its potential to identify underlying themes in the way local communities perceive ill-health, and attempt to manage it. In addition, the current study addresses some of the identified knowledge gaps by carrying out a broad- based study of perceptions of health and influences on health-seeking behaviour, based on information from both the mother and the father. The main research questions addressed here are:

1. Which are the preferred treatment-seeking approaches for parents at the Kenyan coast?
2. What factors influence the decision to go for either modern or traditional healing?
3. Who makes the decision on where and when children should go for treatment?

## Methods

### Ethics Statement

The Kenya Medical Research Institute National Scientific and Ethical Committees approved the study. Written informed consent was obtained from all study participants prior to participation.

### Study Sites

The study was undertaken in 2010 and 2011 at two rural sites at the Kenyan coast: Kilifi and Msambweni. In Kilifi, the study was based at the Centre for Geographic Medicine Research (coast) - KEMRI. The Kilifi district has an estimated population of 719,000 people [15]. Undernutrition is common in Kilifi, as 40% of children under five show anthropometric signs of undernutrition, and 47% present with biochemical markers of iron deficiency [16]. Kilifi District is the second poorest district in Kenya, with more than 67% of the people living below the poverty line, indicating limited access to essential food and non-food items [17]. Most of the people in Kilifi depend on subsistence farming, but frequent rain failure has resulted in insufficient crop yields, compromising food access in the general population. The majority of the population in Kilifi belong to the Mijikenda ethnic group. Two Bantu languages are commonly spoken in the area, Kigiriama (the local vernacular) and Kiswahili (a lingua franca and the national language). In the study area, 47% of the population identify themselves as Christians, 13% Muslim, 24% Traditionalists, 12% ‘other’, and 4% unknown [12]. A significant proportion of the population retain elements of their traditional beliefs and practices, which sometimes guide their health-seeking behaviour [12]. A typical home in Kilifi comprises a large homestead with several small huts built in the compound. In these homes, extended families live together and share in the daily chores such as cooking and fetching water. It is typical for homesteads to have members of three different generations sharing in childrearing duties. The most senior male member of the household (seniority is largely determined by age) is usually considered to be the head of the household.

In Msambweni the study took place within the catchment area of Msambweni District Hospital. Msambweni district is situated at the South coast of Kenya, near the Tanzania border. The district has an estimated population of 283, 658 [18]. The community is rural and depends on crop agriculture as its major source of living. It is estimated that 57% of the population lives below the poverty line. The people belong predominantly to three ethnic groups, with the Digos being the majority, followed by the Durumas. The main language spoken is Kidigo with Swahili as the lingua franca. It is a malaria-endemic area with high rates of malnutrition. Ninety percent of the population is Muslim. The traditional way of life and customary beliefs of the Digo community are quite intact, and people still use traditional healing practices on a day-to-day basis [19]. Similar to Kilifi, it is common to find households with three generations, also consisting of a large homestead with small huts surrounding it, where people share various duties including childrearing and household chores.

### Study Participants and Sampling

This study was carried out as part of a larger study looking at developmental outcomes and parenting behaviour in respect of children less than 18 months of age at the Kenyan coast. In the larger study more than 200 mothers were involved. We looked at health-seeking behaviour because an earlier study indicated that the health-seeking behaviour of parents was closely related to the quality of care-giving. A randomly selected sub-sample of a total of 53 parents and caregivers were involved (table 1 presents a detailed description of the sample). In-depth interviews were held individually with each of these parents and caregivers. Parents attending the Mother-Child Well Clinics were approached for informed consent to join the study during routine post natal visits to the health clinics. Selecting samples from the MCH provides as an opportunity to interview people with access to a hospital as well as traditional medicine. Such a sample allows us to study the decision making process where different alternatives are available. To clarify some of the issues raised during the initial interviews, another randomly selected sample (N = 5, 3 females and 2 males) was involved in the study. Here we largely focussed on a sample likely to give in-depth information on the meaning of indigenous health-related terminology and health-related practices.

**Table 1:** Sample Characteristics.

	Males (N = 23)	Females (N = 30)
**Age**		
Range	20–70	17–75
Mean (SD)	37–43(13.23)	30.23 (12.42)
**Educational Status**		
Unschooled	3 (13.0%)	5 (16.70%)
Primary incomplete	5 (21.7%)	9 (30.0%)
Primary complete	5 (21.7%)	8 (26.7%)
Secondary Incomplete	3 (13.0%)	5 (16.7%)
Secondary complete	3 (13.0%)	1 (3.33%)
Higher educational vocational	1 (4.3%)	-
Higher educational university	3 (13.0%)	2 (6.7%)
**Number of children**		
Range	1–9	1–7
Mean (SD)	3.70(2.42)	2.47 (1.75)

### Data collection

Data was collected using in-depth interviews. Following informed consent procedures, parents were interviewed in a quiet corner away from the presence of other people. All interviews were audio taped. Each interview took approximately one hour. The interviews were guided by a standard set of questions to ensure standardisation. Probes and clarifications were sought as deemed necessary. Parents were asked to express themselves in the language in which they were most comfortable. Sessions therefore ran in both Kiswahili and Kigiriama or Kidigo interchangeably.

### Interview tool

A checklist of questions was developed by the research team through discussion and consensus. Refinement was based upon the initial interviews and discussion of the initial transcripts. Table 2 presents the interview schedule used. The questions presented are core to the interviews; however, probes were introduced to clarify and enhance the quality of the interviews. Moreover, although the main focus of the study was childhood disease, sometimes adults did share their own experiences of health-seeking, and we included the information in cases where it helped to clarify the points raised.

**Table 2:** Interview Schedule.

1. When your child is sick what steps do you take?
2. Do you have a specific person (traditional vs. alternative healer) you go to for treatment?
3. Under what circumstances do you decide to go to the traditional healer or to medical doctors?
4. Who decides on where you should take the child for treatment?
*Example of probes introduced:*
5. What is the purpose of the hirizi? What kind of protection does it afford?
6. What factors have made you not to go for treatment from traditional healers?
7. How did the traditional healer treat you/your child?

### Data Management and Analysis

The final transcripts used for analysis were based on the audio-taped materials. Data was analysed with the assistance of NVIVO 9 software programme according to the framework analysis [20], [21]. The transcripts of the interviews were reviewed and read (familiarisation), during which a coding scheme was developed. The transcripts were coded by creating ‘nodes’ in the NVIVO programme), with each node examined separately. The first author (AA) developed coding schemes and identified themes. The themes identified were then evaluated and checked by one of the authors (JG), who also independently coded five randomly selected transcripts. Themes identified by JG were compared with those identified by AA, with an emphasis on consistency and redundancy. Any disagreements in coding were clarified by consensus. Direct quotes arising from the discussion are presented to support identified themes. Three of the authors - AA, JG and GB - checked for the accuracy of the translations and interpretation of the quotes presented.

## Results

Table 2 presents an overview of all questions asked. The results are presented in five broad themes: *sources of authority in families*; *treatment preferences*; *influencing factors*; *traditional healing*; and *avoidance of traditional medicine*. Quotes and conversational trends are presented to support our identified themes. In these quotes, local terms related to ill-health and treatment-seeking are presented, while no direct translation of these terms are presented in the text (in an effort to avoid changing the respondents' meaning). We also present the meaning of the terms used to describe different diseases, and the closest medical translation of the terminology. In this study, traditional medicine is used to indicate all non-biomedical treatment sought, and these treatments are based on cultural beliefs and practices. Earlier studies indicate that these healing practices are influenced by the different belief systems that are predominant at the Kenyan coast including influences from traditional African belief systems, and Islamic influences. This usage of the term ‘traditional healing’ is in line with the WHO definition [22].

### 1. Preference of treatment

Under this theme, participants in our study indicated that biomedicine (hereby used to refer to both attendance to clinics and home treatment using drugs bought over the counter in shops) was their first preference for treatment. Among our coded responses in this node, we observed that the majority of parents approached a medical doctor to seek treatment or used home treatment compared to those who went first to a traditional healer and those who preferred prayers as their first intervention for a sick child.

*“I monitor them myself first, I check for fever so that before taking them to hospital the fever should not be too high, I get them medicine to take and if their temperature keeps rising I will run to hospital”* [ Mother, 32 ]

*“First we buy medicine, we give it to them and if it does not work we take them to hospital” [Mother, 29].*

Other mothers noted that:

*“When he has fever and flu, I give him paracetamol so as to reduce the fever. And if the temperature is really high, I take a piece of cloth, put it in cold water and wipe the child” [Mother, 31]*

*“First I buy them drugs from the shops then bring them to hospital [if drugs did not help]”* [Mother, 23]

*“Hospital, but if at home we have drugs like paracetamol, we give them and we monitor them”* [Mother, 34]

Despite this obvious preference for biomedicine, many participants still responded that they had also gone to traditional/spiritual healers. Among those we interviewed around a quarter claimed never to have gone for traditional healing. We therefore explored the factors that influence people to go for traditional healing.

### 2. Influencing factors

This theme had the following subthemes:

#### a. Disease perception/attribution of causation

The participants noted two different types of illness which required different treatment or management approaches. Some illnesses were best suited to being treated by the medical doctors, while other diseases were most suited to treatment by traditional healers (Appendix S1). They noted that diseases such as malaria, typhoid and fever caused by ‘natural causes’ could best be treated by biomedicine. When they had such illnesses, they would go to hospital.

*“For hospital it is like coughing, malaria, typhoid, eyes paining, and things like that”.* [Father, 34]

*“When the child has malaria I take them to hospital. Also when they have fever, diarrhea, vomiting - then I take them to hospital….”* [Mother, 25]

Some conditions, particularly those with mental health symptoms such as hallucination or anxiety, appeared to be uniquely suited to traditional healing. The following extracts exemplify the above-mentioned conditions;

*“There are diseases/conditions that are abnormal, take for instance someone who sleeps well and at night gets ill and starts screaming and calling out to people no one else can see, then we decide that hospitals cannot deal with this condition, and take them to experts who can see what the person can see.”* [Mother, 34]

*“Long ago, when I was young, I was taken to a traditional healer… I had lost consciousness; I was seeing things that I could not understand, having nightmares, dreaming I was dying …”* [Father, 34].

*“To have something draw your blood until you become white, or epilepsy, is when we take the child to a traditional healer to treat them.”* [Father, 34].

*“When you feel that something is crawling in your stomach or legs.”* [Mother, 19]

Additionally, illnesses such as ‘chirwa’ or ‘nyuni’ (Appendix S1) which are presumed to have arisen from the breaking of taboos, witchcraft, evil eye and spirit possession were also seen to be best treated by the traditional healers.

*“Like chirwa we take it to a traditional healer, ……..also nyuni we take it to a traditional healer or a teacher (spiritual leader) to read the Quran since people believe it is caused by an evil spirit” [Father, 27]*

#### b. Seeking alternatives

Seeking medical care in clinics and other health centres is the first preference for those seeking treatment for their children. However, in cases where the child's health deteriorates or does not seem to improve, more than half of the parents we talked to, decided to look for other alternatives, such as visiting a traditional healer.

*“When the child gets ill, I will first take them to hospital, until the hospital cannot manage it. When it is impossible [for the hospital to fully treat them] I will wait for the child's father and tell him ‘let us go and try traditional medicine, it is now time to try traditional medicine’………”* [Mother, 50]

Another parent we talked to, noted that when treatment seems not to work, they go for prayers to a faith healer

*“If I have gone to the doctor and received treatment and still see no improvement, then if the doctors have been unable to treat, I will go to seek prayers from a religious leader.”* [Father, 34]

Another participant emphasised the need for traditional healing if the conventional treatment fails.

*“When the hospital is unable to treat the illness then I will go to the traditional healers…”* [Father, 39]

Few of the participants provide a time frame within which they moved from one treatment system to the other. One parent mentioned that

*“It depends on the sickness: personally I will verify the symptoms, when it's a high fever I will give him/her duration of one day before taking him/her to the hospital and during that time I will just buy painkillers just to ease the pain but when the sickness persist then I will take him to the hospital.” [Father, 45]*

#### c. Prevention of ill health

Another important aspect of traditional medicine that was reported by participants is its perceived preventive potential. The parents we talked to noted that even before a child was ill, they took various steps to protect the child from possible ill-health. It was reported that children were taken to traditional doctors to receive protection from various bad occurrences including transgression of taboos, the evil or jealous eye or witchcraft, among others. For instance, a mother explained that if a woman has had an extramarital affair while pregnant, she would go to a traditional healer so that she can receive a potion to protect her unborn baby from getting ‘chirwa’.

*“For instance, if you are pregnant and you go out and have a sexual relationship with a man other than the father of your unborn baby, then you can go to a traditional healer to receive advice before having the baby. There is a potion to be used to wash the baby [usually done after the child has been born]”* [Mother, 22]

Another mother explained the different ways traditional medicine is used as preventive treatment:

*“Right after giving birth, there is something called ‘pande’ we tie on the child's waist, so in case the child's father has a sexual relationship with another woman (i.e. other than the child's mother) and comes to hold the child, the child will not get any disease. The disease is referred to by us as ‘chirwa’ and when the child has been tied to ‘pande’ they won't get chirwa. There is also oil, which you are advised to apply on the child's shoulders going down; this is called ‘kiza’. There are also ‘vipande vipande’ which you tie on the hands as a means of protection from ‘dege’ and ‘dzongo’”* [Mother, 22].

The following participant explains further the conditions that facilitate the use of amulets to protect the child.

*“Diseases we are protected from with amulets include ‘nyuni’ and seizures, among others…….You may hear that a child has been bewitched so that spirits can possess them, but with the right amulet they will be protected.”* [Mother, 29]

#### d. Counselling and advice of elders

In addition, we were told by the participants that parents opted to go for traditional healing based on the counselling and advice given by elders within the family or the community. In the two communities that we worked in, ignoring the counsel of an elderly person is generally frowned upon. Therefore parents would take the child for traditional healing even in cases where they as parents did not strongly feel that their child was likely to receive proper treatment by the traditional healers.

*“Sometimes the grandparents may advise you to take the child to a traditional healer, or an older person may advise you to take the child to hospital, [if you ignore them, it won't be good].”* [Mother, 29]

#### e. It is not really about money

The cost of hospital was rarely mentioned. Our discussions with the mothers indicated that the choice of the kind of treatment was guided more by attribution of aetiology of the disease. For instance, in the discussion on where they seek treatment, a participant pointed out that they would go to the hospital for care if the treatment was free; we then inquired why they would not spend the money they spend at the traditional healer taking the child to hospital. They responded that they were not sure if the hospital had the correct medicine/treatment for ‘nyuni’ or ‘vitsumbakazi’.

*“I would love to go to hospital if it were free, because where there is free treatment I want to go, but there is nothing for free …… I am not sure if the doctors at the hospitals have the correct medication for ‘nyuni’ and ‘vitsumbakazi.”* [Mother, 22]

### 3. Traditional healing

Our participants reported various forms of traditional healing, including the use of herbs, the use of prayers, religious signs and symbols, and rites and rituals. The descriptions below show a religious form of healing

Spiritual healing

*“We use medicine referred to as ‘kombe’….amongst us the healer will take verses from the Quran, write them on a piece of paper, put in a bottle with water and read over that …. The patient is given this (the bottle of water) to use.”* [Mother, 22]

Example of herbal treatment

*“You are given an amulet, and then given another medicine to apply on the forehead. ……..There are herbal roots that you are given to boil, then drink.”* [Mother, 40]

Another participant explained, in a slightly more detailed form, his treatment after being possessed by a spirit and the hospitals could not offer adequate treatment.

*“I had been employed at a construction site. In the evening when I was sleeping, I saw something like a bomb coming to hit me, then I had someone in my sleep telling me to wake up, not sure who but I think it was God's strength (grace), and when I woke up something {that could not be seen} hit my body. I went to the medical doctors; they examined me but they could not identify the problem. When I went to work I would cry and complain [because of pain], but no one could understand. So I went to a traditional healer who said that someone had sent to me a ‘jinii’ (spirit).*

*We looked for chicken, a goat, and some herbal roots. I was given some to use while bathing and the other to drink. The timing for using the medicine was just like the hospital one i.e. morning and evening; and with God's help I now feel OK.”* [Father, 52]

### 4. Avoidance of traditional medicine

As noted earlier, most of our interviewees reported that they used both traditional and biomedicine. However, almost a quarter of those interviewed claimed never to have used any alternative healing at any point in their lives. Given the prevalence of its use in these two communities, we were interested in understanding what made this group of people not go for traditional healing. A common reason given was that traditional healing was found to be out-dated. People expressing this view thought that it was backward and irrelevant for people in the modern world to still go for traditional healing.

*“Traditional healers are there, but the use of traditional healers belongs to the old days, that is why we now have hospitals. When you take your child to a traditional healer, they will pour cold water on them, and make them even more ill.”* [Father, 32]

*“I am civilised and educated, so my decision is to go directly to a medical doctor”* [Father, 34]

For some of the participants, the main reason they did not go for traditional healing was because they did not find it efficient. These interviewees noted that they had observed certain members of the community consistently going for traditional healing, and yet their condition did not improve.

*“It does not heal nor does it prevent ill health, to be honest.”* [Father, 30]

Others reported that they have not been to a traditional healer because their children have not yet experienced any severe ill-health that could not be treated by the hospital.

### 5. Sources of authority

Almost half of the participants had the view that fathers were the ultimate decision-makers in seeking treatment. It was noted that whenever the child required medication or was ill, the child's father was consulted; as noted by one mother

*“It is the father.”* [Mother, 34]

A father's role seems to be very much defined by his status as the breadwinner in the family. It was observed the father had to be consulted so that he could provide money to go for treatment.

*“From my husband….. Where else would I get money?”* [Mother, 50]

These observations by the mothers were confirmed by the fathers:

*“It is the father who makes the decision”* [Father, 32]

Moreover, we inquired from the fathers whom they consulted while making such decisions. This is especially relevant in cases where the child has been undergoing treatment for some time with no signs of improvement; both parents make the decision on when, where and to whom to go and seek medical care from.

*“It is the child's mother, we usually sit down and talk about it, plan on what to do next, since she is the closest person.”* [Father, 36]

Similar sentiments were noted by other participants as is exemplified by the quote below:-

*“It is me and my wife.”* [Father, 34]

The role of the father as the decision-maker may vary as a result of living arrangements. It was observed that when the women were single parents or living away from their husbands, they consulted someone else. As noted by one participant

*“Before one does anything they must first talk to the child's father, if they are living together.”* [Mother, 31]

In cases where the husband was not around, most of the mothers reported consulting their own parents sometimes in-laws, and in rare cases they consulted older neighbours.

## Discussion

Our study had three key findings. Firstly, traditional healing systems coexist with the biomedical system and both complement each other. Secondly, the biomedical system was the preferred treatment but traditional healers are consulted when biomedical system seemed to have failed, and for diseases perceived to have supernatural causes e.g. mental illness. Thirdly, the decision making process on when and where to go for treatment was complex. This process involved various members of the family with fathers being the ultimate decision-makers.

### Traditional vs modern medical approaches

The recognition of biomedical treatment as the first choice of treatment is in line with expected health-seeking behaviour from a Western perspective. The practice of home treatment with drugs largely bought over the counter has been widely reported in Africa. For instance, in a Nigerian study [23] most of the 168 mothers included in the study used some form of home treatment in the first 24 hours of their children's illness. At the same time, the high prevalence of home treatment is a concern. Earlier studies indicated that without intervention, most people receive inappropriate medication or the incorrect dosage, which may contribute to the worsening of the condition or the development of drug resistance. There is evidence that community-based educational interventions can improve patterns of self-medication. A good example is the successful shopkeeper training programme in Kilifi, Kenya [24], [25]. In the shopkeeper programme, rural drug retailers were educated on symptoms of malaria, and learned to advice on selling the right drugs and the correct dosages. Evaluation indicated a significant improvement in the correct usage of anti-malaria drugs during childhood illness by both the retailers and the community.

The decision to go for traditional healing was motivated by two main issues: a) the perceptions of the cause of the illness, and b) the duration, severity and chronicity of the illness. We observed that in the communities in which we worked, diseases had been categorised into two types: the diseases that were taken to hospital, and those that were taken to traditional healers. The diseases not taken to hospital are largely those thought to arise from supernatural causes such as spirit possession, witchcraft and breaching of taboos, among others. From a Western perspective, it also seems that psychological and psychiatric problems are presented to the traditional healers. Our findings are in line with earlier findings from other parts of Africa, that the perception of ill-health in Africa is much more complex than the mainstream biomedical approach. The mainstream model or conceptualisation of health and illness is primarily biological approach. In this model, disease is perceived to be an abnormal physical state caused by bacteria, viruses, hormonal or chemical imbalance in the body [26]. However, in many non-western settings, the conceptualisation of well-being and ill-health is much more holistic, involving the body, the mind and in some instances the supernatural [26]. For example it was observed that among the Bira of Mobala and the Nande of Mukulia, of vthe Democratic Republic of Congo [26], the aetiology of ill-health could be attributed to seven different causes. These ranged from natural causes to transgression of cultural taboos (e.g. eating forbidden food) to witchcraft.

We found that there still exists a high prevalence in the use of traditional medicine; although there were some who observed that it was old-fashioned. According to the WHO 2002 report on traditional medicine, at least 80% of people in Africa use traditional medicine at some point in their lives [22]. These results imply that the efforts to improve health care access in Africa cannot ignore traditional health systems. Of interest here is that our results clearly indicate that traditional healing plays a limited but complementary role to the formal health system. It seems as if these two systems compete at a decision-making level within families, rather than publically competing against each other. Parents opt for one of the forms of treatment and if this does not appear to be working, they opt for the other. The fact that Africa has a severely under-resourced health care system, an approach where modern medicine and traditional health care systems complement each other may be the most efficient and cost-effective way to meet the huge need for health care in the African context. As recommended by the WHO [22], various approaches can be used to encourage integration between modern and traditional health systems. These could include enhancing medical doctors' sensitivity towards the role of traditional healing, and encouraging traditional healers to develop some collaborative work with medical doctors. There are various ways in which traditional healers can complement medical doctors, for example: acting as referral points {sending patients for biomedical treatment}; discouraging traditional healers from any practices that may potentially harm the patients; and encouraging and monitoring adherence to the treatment regimen recommended by medical doctors.

The collaboration between the biomedical and the traditional healing system has to be guided by the need to ensure that the population moves towards optimal health seeking behaviour. Our results indicate that traditional healing in these communities is a mix of various practices including herbalist, spiritualism and practices based on superstition which is consistent with earlier observation from researchers in this community. Previous studies in this area indicate that the key role of traditional healing has been to provide psychosocial and emotional support, an aspect of support that could be significant in helping families cope with chronic illness. Traditional healers were perceived to have better communication skills thus building trust and better communication skills [12], [27]. Experience from elsewhere [28] indicates that a successful collaboration would require sensitivity to ensure the implementation that trust and respect for each other leads to a successful partnership.

Our findings on the relationship between people's beliefs and health-seeking behaviour further support earlier reports on the link between behaviour and health outcomes. As noted by Taffa and Chepngeno [29], such associations between belief and behaviour enhance the possibility of reducing the occurrence of life-threatening illness, and its impact on child development, through the promotion of optimal health-seeking behaviour.

### Decision makers of health seeking behaviour

The decision on when and where to take the child for treatment was ultimately made by the father. This view was held by both parents. Fathers took the ultimate decision based on their positions as head of the family, but also because they were the ones who provided funds for seeking treatment. Our results further emphasize the need to actively involve fathers in health interventions and programmes so that they can make informed decisions on behalf of their families. However, we also observed a much stronger position of women in the decision-making process compared to many of the earlier studies [5]. For instance, many of the fathers said they consulted with their wives when making decisions about treatment for their children. This was confirmed by the women we interviewed. In summary our data show that fathers play a crucial role in the final decision taken. It is therefore important to emphasize that in intervention and educational programmes, the different voices in the decision-making processes should be taken into account.

The pattern of our results indicate that social status, educational levels and power both within the family and larger community has the potential to impact on health seeking behaviour. For instance, some of the participants indicated the use of traditional medicine was outdated and for those with limited education. It would have been interesting to examine these patterns in greater details. However, our sample sizes were too small to allow for this level of analysis. Future studies investigating these effects are recommended.

We interviewed both mothers and fathers as a means of including the father's voice into the discourse. Our pattern of results indicate a high convergence between mothers and fathers in their perceptions of the person who is the decision maker, on where and when to go for treatment, and attribution on causation of illness. This indicates there is a generally accepted cultural pattern of reasoning about health-seeking behaviour in these two communities that may not differ by parental gender.

### Limitations of the study

We used in-depth interviews only in our data collection. Triangulating our results with focus group discussion, observations and surveys could have been used to increase validity of our data. However, the high agreement in our respondents and the patterns of results closely mirroring earlier disease-specific studies (such as those by Kendall-Taylor [11], [12] and Mwenesi [30]) attest to the validity of both our data and our conclusions. Further study of the potential influence of socio-demographic aspects e.g. educational levels and psychological processes, e.g. trust in the biomedical sphere or in the traditional treatments, and concerning the timeframes for decision making (e.g., time taken to move from biomedical to traditional healing and vice versa) is urgently needed to ensure a more detailed understanding of the decision making processes at the individual level.

### Conclusions

Traditional healing systems and biomedical treatment are used as complementary services in these communities. The decision to go to traditional healers is affected by the disease symptoms, the extended family, or the homestead the families live in. Parents differentiate between going to a hospital or to traditional healers, depending upon the complaints and duration of illness of their children. Fathers are found to make the ultimate decisions regarding health-seeking behaviour.

## Supporting Information

Appendix S1 **Table summarizing local terms related to ill-health, causation and treatment approaches as highlighted by participants.**
(DOC)
