# Supplementary material for: Socio-Cultural Determinants of Health-Seeking Behaviour on the Kenyan Coast: A Qualitative Study
Source: PLoS One. 2013 Nov 18;8(11):e71998. doi: 10.1371/journal.pone.0071998 (PMC3832523; doi:10.1371/journal.pone.0071998)
Supplement: Appendix S1 — Table summarizing local terms related to ill-health, causation and treatment approaches as highlighted by participants. (DOC) [file pone.0071998.s001.doc]

**Appendix I: Descriptions of Local Terms Related to Ill-Health, Causation and Treatment Approaches as Highlighted b**y Participants.

| **Disease/ terms** | **Description/ Symptoms and causation** | **Biomedical name** | **Some of the Suggested Treatment approaches** |
| --- | --- | --- | --- |
| Chirwa | Wasting of body, general weakness of body. The child is stunted and does not grow or fails to thrive. Diarrhoea. Excessive thinness. Fatigue/ low energy levels. This is caused when the parents break sexual taboos and even with correct dietary intake the child will not get better; parents must stop breaking the taboos. | Malnutrition | 1. Taking the child away from the parent 2. Cleaning the child with herbs (with leaves from seven specific trees). The child had to use this for 6 consecutive months. 3. Using amulet (see below) |
| Nyuni | Literally translated it means a bird. Refers to a spirit that comes in a bird’s form leading to high fever, unconsciousness, eyes turning white and convulsions. | 1. Febrile convulsions 2. Cerebral malaria | 1. Reading the Quran to the child 2. Should not be injected 3. Peeing on the child chases away the spirit. 4. Bathing the child with herbs mixed with water, especially at a refuse dump |
| Nyago | These are natural spirits inhibiting different areas. A person can be possessed by them either by crossing their habitants or some children having blood types that attract the spirits. Symptoms include high fever, convulsions and seizures. | Epilepsy | Same as Nyuni (see above). |
| Jini | This is a spiritual creature that exists but cannot be seen. They can be good or evil. These are spirits controlled by witches and others. Usually the intent to send a ‘jini’ to a person is to do harm so they are usually considered evil. Someone possessed by a ‘jini’ may show several symptoms including anaemia (the jinni feeds on their blood); stroke and paralysis; severe headaches. Additional symptoms may include self-hatred, suicidal tendencies and general desire for self-harm. One can feel their chests contract as if they are carrying a heavy burden. | Psychosis | 1. Spiritual healing e.g., reading the Quran. 2. Sacrificing an animal, so that the blood can be offered to the spirits. 3. ‘Kombe’ treatment: Quran verses immersed in water are given to people to drink or wash. 4. Severe cases may involve elaborate ceremonies to appease or remove the ‘jinni’ |
| Vitsumbakazi | Spirits believed to move around during the night. Someone who encounters these spirits is likely to show the following symptoms:  Anxiety, being worried, panicking, shouting and screaming for no apparent reason, experiences nightmares, and hallucinations (see or hear sounds only they can hear). | Psychosis | 1. Largely spiritual healing. |
| Kunyonywa damu/ safura | Child becomes pale, paper-white, eyes turn yellow, they become weak and have low blood levels. The skin peels and they swell. Increased heart rate, This syndrome arises because the child is possessed by a spirit which will feed on their blood. | Anaemia/ sickle cell anaemia | 1. Use of rose water 2. Giving the child fresh blood from an animal 3. Important to remove the spirit before undergoing the medical treatment since the spirit will keep sucking on the blood. |
| Dege/dzongo | Evil eye: lustfully desire or evil stares by an adult (towards a child) A child is said to have experienced this if they fall suddenly ill, have a chronic illness or an illness that cannot be explained. | Unknown | 1. Parents often tie amulets on their children’s arms, feet or waist to protect against the evil eye. 2. Apply special oil (coconut oil or sesame oil especially prepared to act as protective oil.). |
| Pande | Amulet made out of special wood. It is usually worn around the arm, leg or waist as a protection. |  | Protects against   1. The evil eye 2. Chirwa (lacking of striving due to breaking of sexual taboos) 3. Witchcraft |
| Hirizi | Amulet made from various materials such as religious readings, wood, and herbs. Also tied around the arm, leg and waist as a protection. |  | Similar to Pande (see above). |
| Mwarobaini | Herbal medicines mainly leaves | Neem products | Against malaria symptoms. |
